# Assessment of Chronic Postsurgical Pain Knowledge Among Surgical Nurses in a Turkish University Hospital

**DOI:** 10.1155/prm/9954969

**Published:** 2025-04-07

**Authors:** Aysel Doğan, Runida Doğan, Dilek Güneş, Nazlıcan Bağci

**Affiliations:** ^1^Faculty of Health Sciences, Department of Surgical Diseases Nursing, Toros University, Mersin, Turkey; ^2^Faculty of Nursing, Department of Surgical Diseases Nursing, İnönü University, Malatya, Turkey; ^3^Faculty of Health Sciences, Department of Nursing, Fırat University, Elazığ, Turkey; ^4^Vocational School of Health Services, Medipol University, Istanbul, Turkey

**Keywords:** chronic postsurgical pain, knowledge, nurse, surgical clinics

## Abstract

**Objective:** Chronic postsurgical pain (CPSP) is a significant yet often underrecognized complication following surgical procedures, impacting patients' quality of life. Surgical nurses play a crucial role in postoperative care and pain management, making their knowledge of CPSP essential for improving patient outcomes. The aim of the study was to assess CPSP knowledge among surgical nurses in a Turkish University Hospital.

**Methods:** This descriptive cross-sectional study included a total of 175 nurses. Data were collected from nurses employed in the surgical units of İnönü University hospital between May 15 and June 15, 2023. Personal information and knowledge-level forms created by the researchers were used for data collection. IBM SPSS Statistics v.25 was used for the statistical analysis.

**Results:** The mean CPSP knowledge score of the nurses working in surgical clinics was 9.26 ± 1.40 (min. = 0, max. = 12) and 75.4% of them had sufficient knowledge. Surgical nurses' knowledge level of CPSP was influenced by their education level (*p* ≤ 0.001) and total duration of employment in the surgical department (*p*=0.002).

**Conclusions:** Although most surgical nurses had sufficient CPSP knowledge, gaps remained, particularly among those with lower education levels and less experience. Targeted training programs and continuous professional development initiatives are recommended to enhance CPSP awareness and improve postoperative pain management practices.

## 1. Introduction

Pain that occurs after surgery is referred to as postsurgical pain (PSP) [1, 2]. Pain experienced within the first 7 days after surgery is described as acute pain, if it lasts longer than 7 days it is termed prolonged pain, and if it persists for more than 3 months it is called chronic PSP (CPSP) [2, 3].

CPSP is a common but underrecognized complication of surgery that can cause functional limitations and psychological distress in patients. It is defined as pain that is localized to the surgical site or a referred area, persists for at least 3 months after surgery, was not present before surgery, or has characteristics or increased intensity different from those of preoperative pain [1]. It has been reported that CPSP, the severity of which may vary depending on the type of surgery, is not associated with postoperative complications [4].

Chronic diseases such as diabetes, migraine, irritable bowel syndrome, fibromyalgia, autoimmune diseases, and peripheral vascular diseases may increase susceptibility to developing CPSP [3, 4]. Pain is more likely to become chronic in patients with severe preoperative pain symptoms and when PSP is not effectively managed [3, 5, 6]. Other factors that may be associated with CPSP include age, sociocultural factors, fear of surgery, expectation of pain, catastrophizing pain, obesity, genetic burden, surgical history, muscle ischemia, surgical technique, nerve damage, and type of analgesia [4].

Although not well documented in the literature, the incidence of CPSP varies. It can develop after both major and minor surgical procedures. Between 5% and 80% of patients are reported to experience CPSP, particularly after surgical procedures that result in nerve damage. The incidence of CPSP is reported to be 30%–81% after limb amputation, 11.5%–47% after inguinal hernia and thoracotomy, and 3%–56.4% after cholecystectomy [1, 2, 4].

Persistent chronic pain after surgery is a significant factor that can prevent individuals from returning to their daily activities and can impact their overall capacity and productivity. CPSP can have a negative impact on a person's overall quality of life, including their ability to return to work. Additionally, it can be extremely costly in terms of health [4, 7] and may also have psychosocial consequences such as depression, anxiety, sleep disturbance, disability, loss of social roles, isolation, and excessive drug use [7, 8].

Considering the clinical and social impacts of CPSP, it is imperative that surgical team members are well educated and informed about how to prevent it [1, 4, 5]. Nurses, who are essential members of the surgical team, are responsible for many interventions, such as providing perioperative analgesia, promoting venous return, and facilitating early mobilization, which effectively prevents CPSP [4, 9]. Preventing CPSP through competent nursing care in the preoperative and early postoperative period is even more critical, especially given the clinical consequences of CPSP. Therefore, nurses should have sufficient knowledge about CPSP [4, 7–9]. Although nurses' knowledge levels about pain has been examined in many studies [5, 9–11] their knowledge of CPSP has not been investigated. Therefore, the present study was conducted to assess the level of knowledge of nurses working in surgical units regarding the management of CPSP. The study is new and unique in terms of its purpose.

## 2. Materials and Methods

### 2.1. Study Design

This was a descriptive cross-sectional study. It was conducted in line with the Strengthening the Reporting of Observational Studies in Epidemiology (STROBE) guideline.

### 2.2. Study Area

The study was conducted at İnönü University Turgut Özal Medical Center between May 15 and June 15, 2023. It contains 39 operating rooms (27 in the main building and 12 in the Liver Transplant Hospital), 317 intensive care beds (265 in the main building, 36 in the Liver Transplant Hospital, and 16 in the Oncology Hospital), 217 beds in the Oncology Hospital, 152 beds in the Liver Transplant Hospital, and 1585 beds in the main building, including day beds and intensive care. There are a total of 265 nurses working in the surgical units.

### 2.3. Target Population

The study population consisted of nurses who were working in the surgical units of the hospital.

### 2.4. Inclusion Criteria

The inclusion criteria were nurses who provided direct patient care to patients in the surgical unit of the hospital and consented to participate in the study.

### 2.5. Exclusion Criteria

Nurses were excluded from the study if they were on annual or sick leave during the data collection period or if they submitted incomplete survey forms.

### 2.6. Sample Size and Sampling Procedure

The sample of the study was determined as 162 nurses with the assumption of 1–*β* = 0.95 power, *α* = 0.05 error level, and effect size *f* = 0.26 with the program G^∗^ Power 3.1.9.2 [12].

A 10% attrition rate was added, resulting in a final sample size of 178. A simple random sampling technique was employed to select the respondents. This approach ensured that all respondents had an equal chance of selection, thereby minimizing selection bias.

### 2.7. Data Collection Tool

The researchers referred to previous studies when developing the personal information and CPSP knowledge forms to collect data.

#### 2.7.1. Personal Information Form

This form consisted of eight questions: sex, age, marital status, education level, work experience, surgical department in which they worked, total working time in the surgical department, and status of receiving education about CPSP [1, 3, 11, 13–17].

#### 2.7.2. CPSP Knowledge Form

This form was developed by the researchers with reference to previous studies [1, 3, 11, 13–16, 18]. The form consists of 12 statements to which the responses are “true,” “false,” or “no idea.” Seven of the statements are correct and five are incorrect. Each correct response earns 1 point. Wrong and “no idea” responses receive 0 points. The highest possible score is 12. A high score indicates a high CPSP knowledge level. Nurses who received 1–4 points were considered to have insufficient knowledge, those who received 5–8 points were considered to have moderate knowledge, and those who received 9–12 points were considered to have sufficient knowledge.

### 2.8. Validity and Reliability

The CPSP knowledge form was sent to four clinical nurses with a Master's degree in surgical diseases, five surgical diseases faculty members, and two anesthesiologists (11 in total). These experts were asked to provide their opinions on the suitability of the content, as well as the comprehensibility and difficulty level of the questions. All eleven members of the expert committee rated each question/item in the form in terms of its relevance to the underlying construct using a 4-point ordinal scale (1: not relevant; 2: somewhat relevant; 3: quite relevant; 4: highly relevant). Item-level (I-CVI) and scale-level (S-CVI) content validity indices were calculated to measure content validity [19–21].

The item-level content validity (I-CVI) was computed for each item as the number of experts giving a rating of either 3 or 4 divided by the total number of experts. The scale-level content validity (S-CVI) was calculated as the average of the I-CVIs for all items on the scale. An I-CVI higher than 0.78 was considered excellent, and a minimum S-CVI of 0.80 was deemed acceptable; hence, no items were removed from the form. For reliability, correct responses were assigned a score of ‘1' and incorrect or ‘no idea' responses a score of ‘0.' The Kuder–Richardson 21 (KR-21) formula was applied, yielding a reliability coefficient of 0.771, indicating good internal consistency [22–24].

### 2.9. Data Collection Procedure

The data were collected by the co-researcher through face-to-face interviews between May 15 and June 15, 2023. She visited nurses in the wards where they worked and provided information on the study. She then obtained written consent from the nurses who agreed to participate and were not on annual or sick leave on the data collection dates. She asked the nurses questions from the personal information and CPSP forms and then recorded the answers. Each interview lasted approximately 10 to 15 min.

### 2.10. Data Analysis

The data were analyzed using IBM SPSS Statistics v.25. They were subjected to the Kolmogorov–Smirnov test and were found to not conform to a normal distribution. In the analysis of individual characteristics, descriptive values (percentage, arithmetic mean, standard deviation, minimum, and maximum) and Mann–Whitney *U* and Kruskal–Wallis tests were used to compare the independent groups. Dunn's multiple comparison test was conducted to determine which group was statistically significantly different at a given α level when comparing independent groups. The significance level was set at *p* < 0.05.

### 2.11. Ethical Considerations

Ethics committee and institutional approval were obtained for the study (Date 02.05.2023/Decision Number: 2023/4591). Verbal and written informed consent were obtained from the participating nurses. The principles of the Declaration of Helsinki were adhered to at every stage, from the execution of the study to publication.

## 3. Results

### 3.1. Sociodemographic Characteristics

A total of 175 surgical nurses were included in the study, resulting in a 98.3% response rate. Among them, 72% were females, 44% were between the ages of 21 and 30 years, and 64.6% were single. About 76% had an undergraduate degree, 34.3% had been working as a nurse for 6 to 11 years, 13.7% had been working in an organ transplant clinic, 35.4% worked the surgical department for 13 to 60 months. Moreover, all nurses (100%) had not received any training on CPSP (Table 1).

### 3.2. Knowledge of CPSP

**Figure 1:**
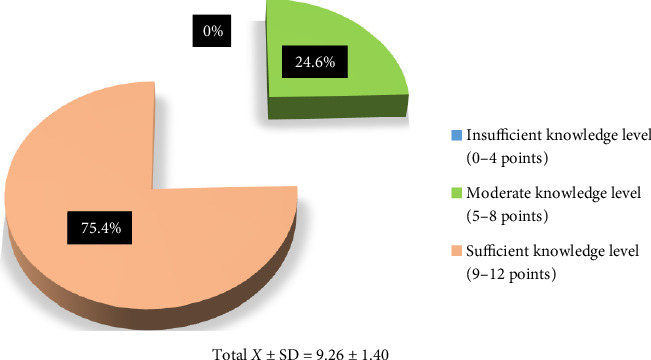
Nurses' chronic postoperative pain knowledge levels.

The mean CPSP knowledge score of the surgical nurses was 9.26 ± 1.40, and 75.4% had sufficient knowledge about CPSP (Figure 1). Analysis of the responses revealed that 84.6% correctly responded to the statement “In cases where PSP is not well managed, the pain is likely to become chronic,” 83.4% correctly responded to “Depression and anxiety have no effect on the development of CPSP,” 83.4% knew that the statement “Depression and anxiety have no effect on the development of chronic postoperative pain” was false. 78.9% correctly responded to “Surgical fear, pain expectation, and catastrophizing pain do not play a role in the development of CPSP,” and 75.4% correctly answered The surgical technique used has no effect on the development of CPSP (Table 2).

### 3.3. Differences in Knowledge of CPSP With Regard to Sociodemographic Information

A statistically significant difference in surgical nurses' CPSP knowledge was observed in relation to their education level and duration of employment (*p* < 0.05). Post hoc analysis revealed that nurses with an undergraduate degree (*M* = 9.25, SD = 1.34) had higher CPSP knowledge than those with a high school or associate degree (*M* = 8.54, SD = 1.55, *p* < 0.001). Additionally, nurses with a master's degree (*M* = 10.27, SD = 0.95) demonstrated greater CPSP knowledge than their counterparts with an undergraduate degree (*M* = 9.25, SD = 1.34, *p* < 0.001). Regarding duration of employment, nurses who had worked in the surgical department for 121–180 months (*M* = 10.12, SD = 0.95) exhibited higher CPSP knowledge than those who worked 13–60 months (*M* = 9.64, SD = 1.50, *p*=0.009). Similarly, nurses who worked 13–60 months (*M* = 9.64, SD = 1.50) had higher CPSP knowledge than counterparts who worked less than 12 months (*M* = 8.82, SD = 1.44, *p*=0.002) (Table 3).

## 4. Discussion

The study assessed CPSP knowledge among surgical nurses in a Turkish University Hospital.

The majority of the nurses knew that CPSP is more likely if postoperative pain is not managed well. This is consistent with previous studies [1, 4, 25] which reported that postoperative pain can become chronic if not managed well. Thapa and Euasobhon reported that poorly managed acute pain is the most related factor in the development of CPSP [1]. The fact that many nurses recognize the importance of effective PSP management is important for the specification of nursing care interventions to prevent the chronicization of pain and to achieve better patient outcomes. Since inadequate postoperative pain control increases the risk of CPSP, reinforcing this knowledge through continuous education and training could further enhance patient outcomes.

A significant number of participants knew that depression and anxiety had an effect on the development of CPSP. This result is consistent with the literature indicating that depression and anxiety are important factors in the development of CPSP. Hinrichs-Rocker, et al. reported that psychological factors such as depression showed a possible correlation in their study on psychological predictors and correlates for CSPS [26]. Thapa and Euasobhon reported that psychological recognition of patients and taking preoperative precautions have a preventive role in the development of CPSP [1]. In this context, the fact that most of the nurses had sufficient knowledge about the psychological dimension of CPSP can be considered a positive result.

It was observed that majority of the nurses correctly knew that CPSP was not related to the size of the surgery and many of them knew that the severity of CPSP varied according to the type of surgery. This result is consistent with previous studies in the literature in which the effect of operation size and type on the development of CPSP was examined [4, 25]. This finding is encouraging in terms of suggesting that the majority of surgical nurses practice effective postoperative pain management regardless of the type and extent of surgery.

Furthermore, mostly of the participants knew that surgical fear, pain expectation, and catastrophic pain play a role in the development of CPSP. This is in line with the work of Thapa and Euasobhon, who reported that CSPS is related to surgical fear, pain expectancy, and pain catastrophizing [1]. Nurses need to identify psychologically vulnerable patients in the preoperative period, inform them, and encourage them to receive psychological support when necessary to prevent CSPS. The results of the study are valuable in terms of nurses' roles in preventing CPSP.

In addition, the majority of the participants correctly recognized that some chronic diseases increase susceptibility to the development of chronic postoperative pain. Results are in line with the studies of Ramajaki et al., who reported that the development of CPSP is associated with diabetes, and Kraychete et al., who reported that it is associated with many chronic diseases such as autoimmune diseases and peripheral vascular diseases [4, 27]. This result shows that most of the participants had accurate knowledge about this issue. Knowing which patients are at risk of CPSP will be beneficial in the prevention of CPSP by affecting the care and education of patients in that group.

Of the nurses participating in this study, a significant most thought that the surgical technique used affects the occurrence of CPSP. This result is consistent with the Reddi and Lyra et al.'s studies reporting that the surgical technique used is an important factor in the development of chronic postoperative pain [28, 29]. This result is encouraging as it suggests that surgical nurses' understanding of the factors that increase the risk of developing CSPS may empower them to deliver more effective preventive care.

The majority of the participants correctly defined CPSP as pain lasting longer than 3 months in the postoperative period. Although it is a recent addition to the literature [1, 30]. The fact that CPSP is largely defined correctly is a pleasing result in terms of suggesting that nurses follow the newer literature.

More than half of the nurses knew that the type of analgesia was a factor in the development of CPSP. This result is consistent with previous studies reporting the effect of analgesia type on the development of CPSP [4, 25]. Although a large proportion of nurses have accurate knowledge on this subject, all nurses are expected to have complete, accurate knowledge on this subject in order to provide effective analgesia management. Therefore, it is thought that it is necessary to encourage nurses to take professional development courses and specialty training where their knowledge will be updated.

The results showed that the level of CPSP knowledge increased with the level of education (Table 3). According to the post-hoc analysis, the group with the highest level of knowledge on the subject was that with master's and doctorate level education. When the literature is examined, it is seen that the educational status of nurses increases the level of knowledge in many examples in which the knowledge status of nurses on various subjects is examined [15, 31]. The results of the current study are also important in terms of showing once again the benefits of postgraduate education and specialization in nursing.

The results showed that the level of knowledge of CPSP increased as the amount of time spent working in the surgical department increased (Table 3). In studies conducted with different groups, it was found that experience increased the level of knowledge, similar to this finding [11, 13, 32]. This result once again emphasized the importance of professional experience. It shows that experience is an important factor to be taken into consideration when recruiting nurses for surgical units.

When 1 point was given for each statement responded to correctly in the knowledge form and 0 points were given for the other responses, the mean knowledge score of the nurses working in surgical clinics about CPSP was sufficient (Figure 1). This can be considered a good result. Although there are no studies examining the knowledge of nurses about CPSP, there are many examining the level of knowledge of nurses about pain. In a study conducted in a geriatric hospital in Vietnam, it was found that a large proportion of the nurses had insufficient knowledge about pain management, whereas in the study by Alnajar et al. it was found that oncology nurses had sufficient knowledge about cancer pain [14, 33]. It is thought that sociodemographic characteristics such as educational status, working experience, and hospital qualifications may have affected the study results.

## 5. Conclusions and Recommendations

The nurses working in surgical clinics were found to have sufficient knowledge about CPSP. The educational level and years working in the surgical clinic were associated with CPSP knowledge. Although most statements were responded to correctly, some participants had insufficient knowledge. An essential prerequisite for effective nursing care is sufficient knowledge and proficiency. Based on the results obtained from our study, organizing postgraduate training programs for nurses regarding CPSP is recommended. It has been shown in many studies that participation in training programs positively affects knowledge [11, 14, 31–33]. Further studies with larger sample sizes are required to obtain more accurate results. It is recommended that the study be repeated with a larger sample group and that experimental studies be conducted on the subject.

## 6. Limitations of the Study

The first limitation that cannot be overlooked is that the study was conducted at a single center. The second limitation is that this country's university hospitals are considered educational institutions. Thus, nurses working in university hospitals have more opportunities to access information and conduct research compared to those in public and private hospitals. Therefore, our results may differ from those obtained in similar studies conducted at other types of hospital.

## Figures and Tables

**Table 1:** Sociodemographic characteristics of the nurses working in surgical units (*n* = 175).

		*n*	%	$\overset{¯}{X}$ ± SD	Min.–max.
Sex	Female	126	72.0		
	Male	49	28.0		

Age	21–30 age group	77	44.0		
	31–40 age group	53	30.3	34.48 ± 8.45	21–53
	41–53 age group	45	25.7		

Marital status	Married	113	64.6		
	Single	62	35.4		

Education (graduated from)	High school and associate degree	24	13.7		
	Undergraduate degree	133	76.0		
	Master's and PhD degree	18	10.3		

Duration of employment in nursing	1–5 years	48	27.4		
	6–11 years	60	34.3		
	12–17 years	23	13.1		
	≥ 18 years	44	25.1		

Department worked in	Cardiovascular surgery clinic	8	4.6		
	Burn unit	6	3.4		
	Eye-plastic surgery clinic	10	5.7		
	General surgery clinic	18	10.3		
	Ear, nose, and throat clinic	7	4.0		
	Urology clinic	5	2.9		
	Organ transplant clinic	24	13.7		
	Orthopedics clinic	13	7.4		
	Pediatric surgery clinic	12	6.9		
	Thoracic surgery clinic	11	6.3		
	Brain surgery clinic	12	6.9		
	Cardiovascular surgery intensive care	12	6.9		
	General surgery intensive care	16	9.1		
	Organ transplant intensive care	21	12.0		

Total working time in the surgical department (months)	≤ 12 months	45	25.7		
	13–60 months	62	35.4		
	61–120 months	42	24.0	64.37 ± 63.73	2–300
	121–180 months	16	9.1		
	≥ 181 months	10	5.7		

Received education on chronic postsurgical pain before	Yes	0	0		
	No	175	100		

Total		175	100.0		

**Table 2:** Chronic postsurgical pain knowledge of the nurses working in surgical units.

	Nurses' responses		
	True	False	No idea
	*n* (%)	*n* (%)	*n* (%)
1. Pain lasting longer than 3 months in the postsurgical period is described as “chronic postsurgical pain”	130 (74.3)	8 (4.6)	37 (21.1)
2. The surgical technique used has no effect on the development of postsurgical chronic pain	0 (0)	132 (75.4)	43 (24.6)
3. The severity of chronic postsurgical pain varies according to the type of surgery	135 (77.1)	10 (5.7)	30 (17.2)
4. People with severe preoperative pain symptoms are more likely to have chronic pain	85 (48.6)	33 (18.9)	57 (32.6)
5. Chronic postsurgical pain is not associated with surgical complications	6 (3.4)	124 (70.9)	45 (25.7)
6. Chronic postsurgical pain is a problem that occurs only after major surgery	1 (0.6)	144 (82.3)	30 (17.1)
7. Some chronic diseases may increase the predisposition to the development of chronic postsurgical pain	136 (77.7)	6 (3.4)	33 (18.9)
8. Surgical fear, pain expectation, and catastrophizing pain do not play a role in the development of chronic postsurgical pain	0 (0)	138 (78.9)	37 (21.1)
9. Chronic postsurgical pain is experienced especially after surgical procedures that cause nerve damage	106 (60.6)	17 (9.7)	52 (29.7)
10. Depression and anxiety have no effect on the development of chronic postsurgical pain	0 (0)	146 (83.4)	29 (16.6)
11. In cases where postsurgical pain is not well managed, the pain is likely to become chronic.	148 (84.6)	9 (5.1)	18 (10.3)
12. Type of analgesia is not a factor in the development of chronic postsurgical pain	0 (0)	122 (69.7)	53 (30.3)

*Note:* True items are items 1, 3, 4, 5, 7, 9, and 11. False items are items 2, 6, 8, 10, and 12.

**Table 3:** Comparison of the mean chronic postsurgical pain knowledge scores according to the sociodemographic characteristics of the nurses working in surgical units.

		$\overset{¯}{X}$ ± SD	Mean rank	^∗^ *p* value
Sex	Female	9.19 ± 1.42	85.35	0.253
	Male	9.44 ± 1.32	94.83	

Age	21–30 age group	9.24 ± 1.50	88.11	0.964
	31–40 age group	9.32 ± 1.25	89.15	
	41–53 age group	9.22 ± 1.41	86.46	

Marital status	Married	9.19 ± 1.34	85.20	0.310
	Single	9.38 ± 1.49	93.10	

Education (graduated from)	High school and associate degree (A1)	8.54 ± 1.55	63.98	**≤ 0.001**
	Undergraduate degree (A2)	9.25 ± 1.34	87.37	A1-A2/≤ 0.001
	Master's and PhD degree (A3)	10.27 ± 0.95	124.67	A2-A3/≤ 0.001

Duration of employment in nursing (years)	1–5 years	9.35 ± 1.50	92.81	0.331
	6–11 years	9.28 ± 1.45	87.90	
	12–17 years	8.78 ± 1.20	71.09	
	≥ 18 years	9.38 ± 1.29	91.73	

Department worked in	Cardiovascular surgery clinic	8.87 ± 1.80	72.56	0.088
	Burn unit	8.66 ± 2.25	77.33	
	Eye-plastic surgery clinic	9.30 ± 1.41	87.30	
	General surgery clinic	9.11 ± 1.13	81.22	
	Ear, nose, and throat clinic	10.14 ± 1.21	120.07	
	Urology clinic	8.80 ± 1.09	69.20	
	Organ transplant clinic	9.45 ± 1.41	94.92	
	Orthopedics clinic	9.15 ± 1.77	87.81	
	Pediatric surgery clinic	8.33 ± 1.43	57.00	
	Thoracic surgery clinic	9.63 ± 0.92	99.91	
	Brain surgery clinic	10.08 ± 1.31	115.88	
	Cardiovascular surgery intensive care	9.91 ± 1.08	114.67	
	General surgery intensive care	8.93 ± 1.18	72.91	
	Organ transplant intensive care	9.09 ± 1.30	80.88	

Department worked in	Surgical clinics	9.26 ± 1.46	88.56	0.808
	Surgical intensive care units	9.24 ± 1.25	86.55	

Total duration of employment in the surgical department (months)	≤ 12 months (A1)	8.82 ± 1.44	103.54	**0.002**
	13–60 months (A2)	9.64 ± 1.50	72.74	
	61–120 months (A3)	9.21 ± 1.17	84.52	A1-A2/0.014 A2-A4/0.009
	121–180 months (A4)	10.12 ± 0.95	118.56	
	≥ 181 months (A5)	9.10 ± 1.19	78.35	

*Note:* $\overset{¯}{X}$ = mean. Z = Mann–Whitney *U* test. KW = Kruskal–Wallis test.

Abbreviation: SD, standard deviation.

^∗^ *p* < 0.05 is statistically significant.

## Data Availability

The data that support the findings of this study are available from the corresponding author upon reasonable request.
